# Significant Suppression of Non-small-cell Lung Cancer by Hydrophobic Poly(ester amide) Nanoparticles with High Docetaxel Loading

**DOI:** 10.3389/fphar.2018.00118

**Published:** 2018-02-28

**Authors:** Xing Chen, Lili Zhao, Yang Kang, Zhiyu He, Fei Xiong, Xiang Ling, Jun Wu

**Affiliations:** ^1^Key Laboratory of Sensing Technology and Biomedical Instrument of Guangdong Province, School of Engineering, Sun Yat-sen University, Guangzhou, China; ^2^Digestive Endoscopy Center, Jiangsu Province Hospital, Nanjing, China; ^3^Key Laboratory of Mountain Ecological Restoration and Bioresource Utilization, Chengdu Institute of Biology, Chinese Academy of Sciences, Chengdu, China

**Keywords:** cancer, docetaxel, hydrophobicity, nanoparticle, poly(ester amide)

## Abstract

Non-small-cell lung cancer (NSCLC) accounts for over 85% of clinical lung cancer cases, which is the leading cause of cancer-related death. To develop new therapeutic strategy for NSCLC, a library of L-phenylalanine-based poly(ester amide) (Phe-PEA) polymers was synthesized and assembled with docetaxel (Dtxl) to form Dtxl-loaded Phe-PEA nanoparticles (NPs). The hydrophobic Phe-PEA polymers were able to form NPs by nanoprecipitation method and the characterization results showed that the screened Dtxl-8P4 NPs have small particle size (∼100 nm) and high Dtxl loading (∼20 wt%). *In vitro* experiments showed that Dtxl-8P4 NPs were rapidly trafficked into cancer cells, then effectively escaped from lysosomal degradation and achieved significant tumor cell inhibition. *In vivo* results demonstrated that Dtxl-8P4 NPs with prolonged blood circulation could efficiently deliver Dtxl to A549 tumor sites, leading to reduced cell proliferation, block metastasis, and increase apoptosis, then persistent inhibition of tumor growth. Therefore, Phe-PEA NPs are able to load high amount of hydrophobic drugs and could be a promising therapeutic approach for NSCLC and other cancer treatments.

## Introduction

Being ranked the majority of lung cancer, non-small-cell lung cancer (NSCLC) causes human death due to its relative insensitiveness to chemotherapy (Jemal et al., 2008; Umar et al., 2012). More than 60% of NSCLC patients are diagnosed to have advanced or metastatic tumors, which are unsuitable for surgical resection with curative intent (Tong, 2006; Pao and Chmielecki, 2010). Thus, alternative therapeutic platforms to control or inhibit tumor development are highly desired. Docetaxel (Dtxl) is of the chemotherapy drug class taxane, structurally similar to paclitaxel, but more effective as the inhibitor of microtubule depolymerization (Bissery, 1995). In the past decades, Taxotere has emerged as one of the most important cytotoxic agents, with proven clinical efficacy against many cancers including NSCLC (Kintzel et al., 2006; Baker et al., 2009). However, the use of Dtxl in this formulation with non-ionic surfactant Tween 80 and 13% ethanol leads to several well-known adverse reactions due to either the agents itself (e.g., neutropenia, anemia, nephrotoxicity, neurotoxicity, and musculoskeletal toxicity) or the solvent system (e.g., hypersensitivity and fluid retention) (Persohn et al., 2005). Side effects of commercial Taxotere have considerably overshadowed its clinical application.

Nanoparticles (NPs) have been extensively reported for their prominent superiorities that can be delivered to specific sites by size-dependent passive targeting (Matsumura and Maeda, 1986; Pan et al., 2011a,b, 2012; Bertrand et al., 2014; Deng et al., 2016; Lu et al., 2016; Zhao et al., 2016; Li et al., 2017; Lin et al., 2017; Liu et al., 2017; Shi et al., 2017). Among them, numerous nanoplatforms have been utilized to deliver Dtxl for improved cancer treatment (Tao et al., 2014; Bowerman et al., 2017; Laura et al., 2017). However, the clinical application of most reported platforms is hindered by the low loading capacity of Dtxl (Guo and Huang, 2014). Thus, the carrier material with reasonable hydrophobicity is urgently needed (Chu et al., 2013). Amino acid-based poly(ester amide) polymers with both ester and amide blocks on their backbones have been widely studied over many years (Wu et al., 2012a,b; Wu and Chu, 2013), as they possess not only good biodegradability and biocompatibility but also tunable physicochemical properties, especially hydrophobicity (Wu et al., 2011, 2015; Yu et al., 2014), which may be promising for developing NPs with high Dxtl loading.

**FIGURE 1:**
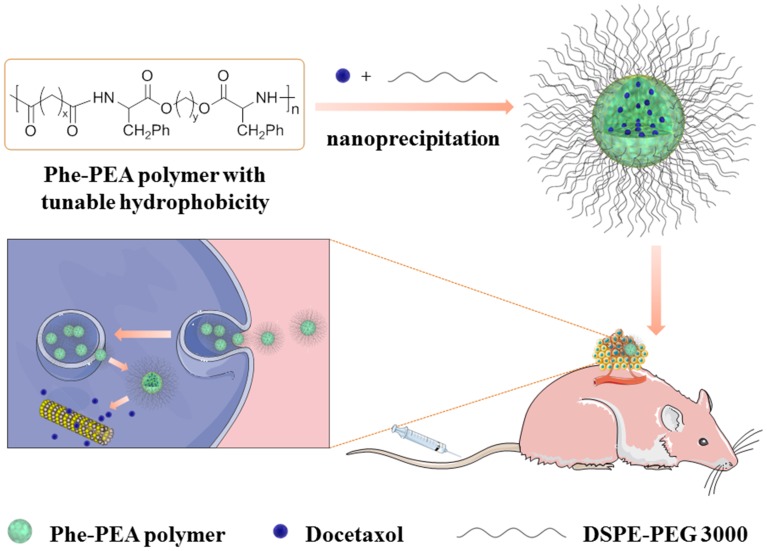
Representative scheme for preparation of Dtxl-8P4 NPs and introduction of *in vivo* and intracellular delivery.

Hence, we postulated that the novel design of hydrophobic L-phenylalanine-poly(ester amide) (Phe-PEA) polymer NPs with higher Dtxl loading could bring about more effective antitumor efficiency with better *in vivo* tolerance. In this paper, Phe-PEA polymers comprised of phenylalanine, diacid, and diol were synthesized and used because of their excellent NP formation and drug loading capability. Dtxl-loaded Phe-PEA polymer NPs were prepared by nanoprecipitation and the physicochemical characteristics were determined. After optimization, Dtxl-8P4 NPs with attractive uptake kinetics and strong cytotoxicity were found to greatly improve circulation retention, then enhance therapeutic effects for A549 tumors with less systemic toxicity (**Figure 1**).

## Materials and Methods

### Materials and Reagents

L-Phenylalanine, 1,4-butanediol, 1,6-hexanediol, adipoyl dichloride, sebacoyl dichloride, toluene-4-sulfonic acid monohydrate, and *p*-nitrophenol were purchased from Sigma–Aldrich and used without further purification. DMSO, acetone, toluene, triethylamine, ethyl acetate, and methanol were purchased from Aladdin. Dtxl was purchased from LC Laboratories. Taxotere^®^ was purchased from Sanofi Aventis. Dil was purchased from Thermo Fisher Scientific, United States. 1,2-distearoyl-*sn*-glycero-3-phosphoethanolamine-*N*-[methoxy-(polyethylene glycol)-3000] (ammonium salt) (DSPE-PEG 3000) was purchased from Avanti Polar Lipids. F-12K, EMEM, trypsin-EDTA, fetal bovine serum (FBS), penicillin–streptomycin solution, phosphate-buffered saline (PBS), and water were provided by Gibco^®^.

### Synthesis of Monomers and Polymers

**FIGURE 2:**
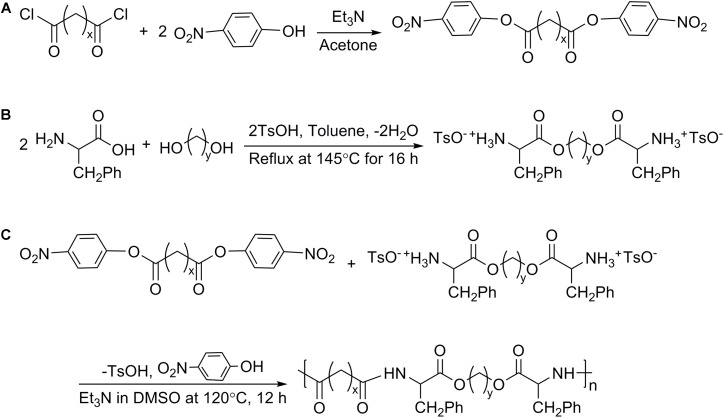
Synthetic routes of Phe-PEA polymers.

The synthesis of Phe-PEA polymers was divided into following steps (Katsarava et al., 1999): (i) preparation of di-*p*-nitrophenyl esters of dicarboxylic acid (monomer I) by condensation reaction; (ii) preparation of toluene-4-sulfonic acid salts of bis(Phe) alkylene diesters (monomer II) via solid–liquid reaction; (iii) preparation of Phe-PEA polymers through solution polycondensation (**Figure 2**). Synthetic details of monomers I and II could be found in previous reports (Wu and Chu, 2012), while Phe-PEA polymers (yield > 80%) were obtained by optimized protocols (Fonseca et al., 2014): monomers I (5 mmol) and II (5 mmol) in dry DMSO (8 ml) were mixed well by vortex and kept at 120°C under vigorous stirring, then triethylamine (15 mmol) was drop-wisely added to get a uniform yellow solution. The mixture was kept at 80°C overnight without stirring and resulting polymers were precipitated by adding cold ethyl acetate, washed with methanol, and dried under vacuum.

Here, two kinds of monomers I were prepared: di-*p*-nitrophenyl adipate (N4, *x* = 4) and di-*p*-nitrophenyl sebacate (N8, *x* = 8). Two kinds of monomers II were prepared: toluene-4-sulfonic acid salts of bis(Phe) butane diesters (Phe-4, *y* = 4) and toluene-4-sulfonic acid salts of bis(Phe) hexane diesters (Phe-6, *y* = 6). Phe-PEA polymers (*x*-Phe-*y*) were prepared by solution polycondensation of monomers I and II at various combinations and summarized in **Table 1**, where *x* was the numbers of methylene in diacid and *y* was the numbers of methylene in diol. Chemical structures of above monomers and polymers were confirmed by ^1^H-NMR (Avance III, Bruker, Switzerland). All the spectra were the same as previously reported (**Supplementary Figure S1**) (Katsarava et al., 1999).

**Table 1:** Phe-PEA polymers and their corresponding NPs.

Monomer I	Monomer II	Phe-PEA polymers	Formed NPs	Particle size (nm)	Polydispersity index	Zeta potential (mV)	Dtxl loading (%)
N4	Phe-4	4-Phe-4	Dtxl-4P4 NPs	154.4 ± 2.7	0.253 ± 0.013	-23.56 ± 2.05	6.7 ± 0.7
N4	Phe-6	4-Phe-6	Dtxl-4P6 NPs	128.6 ± 5.6	0.133 ± 0.005	-26.10 ± 1.13	7.5 ± 0.6
N8	Phe-4	8-Phe-4	Dtxl-8P4 NPs	86.2 ± 0.5	0.125 ± 0.029	-26.39 ± 1.54	8.9 ± 0.9
N8	Phe-6	8-Phe-6	Dtxl-8P6 NPs	67.1 ± 1.2	0.220 ± 0.016	-28.92 ± 0.77	8.5 ± 1.0


For measuring of molecular weight (MW) of Phe-PEAs, gel permeation chromatography (GPC) method was used and the PEA solutions were prepared at a concentration of 1 mg/ml in a tetrahydrofuran (THF) solution. The MWs of Phe-PEAs were determined from a standard curve generated from polystyrene standards with MWs ranging from 841.7 to 2.93 kDa that were chromatographed under the same conditions as the samples. The standard curve was generated from a third-order polynomial fit of the polystyrene standard MWs (**Table 2**).

**Table 2:** Molecular mass characteristics of Phe-PEA polymers.

PEA	Mn (10^3^ Da)	Mw (10^3^ Da)	PDI
4-Phe-4	19.2	23.3	1.21
4-Phe-6	18.7	24.9	1.33
8-Phe-4	22.4	26.8	1.20
8-Phe-6	21.7	27.0	1.24


### Preparation and Characterization of Nanoparticles (NPs)

Dtxl-8P4 NPs were prepared by nanoprecipitation method: 6 mg of 8-Phe-4 polymer and a certain amount of Dtxl (10, 20, or 30 wt% of NPs) was dissolved in 0.2 ml of DMSO. Next, the mixture was dropwise added to 10 ml of aqueous solution containing DSPE-PEG 3000 (20 wt% of NPs) under vigorously stirring. The remaining free molecules and organic solvent were removed by washing with PBS twice using Amicon Ultra-15 centrifugal filters (MWCO 100 KDa, Millipore, United States). Finally, Dtxl-8P4 NPs were dispersed in 1.0 ml of PBS for further use. 8P4 NPs were prepared without Dtxl and used as blank control. Dtxl-4P4, Dtxl-4P6, or Dtxl-8P6 NPs were prepared by the similar procedure using 6 mg of 4-Phe-4, 4-Phe-6, or 8-Phe-6 polymer and fixed feeding of Dtxl (10 wt% of NPs). Dil-8P4 NPs were prepared by mixing pre-determined amounts of 8-Phe-4 polymer, Dtxl, and Dil (3 wt% of NPs) in DMSO, then following above nanoprecipitation procedure. Particle size and zeta potential of NPs were measured by dynamic light scattering (DLS, Zetasizer Nano-ZS90, Malvern, United Kingdom). Morphology of NPs was visualized by transmission electron microscopy (TEM, Tecnai G2 Spirit, FEI, United States).

Docetaxel loading capacity of NPs was determined by Agilent 1260 HPLC with a ZORBAX Extend-C18 column at the temperature of 30°C and a flow rate of 1.0 ml/min (mobile phase, water:acetonitrile = 50:50). The injection volume was 20 μl for each sample. The UV detection wavelength was 232 nm and Dtxl loading was calculated using following equation:

$$\text{Loading capacity\%=}\frac{\text{Weight of loaded drugs}}{\text{Weight of polymers+Weight of loaded drugs}}\times 100.$$

### *In Vitro* Release Profiles

Dtxl-8P4 NPs were transferred to dialysis bags (MWCO 3500 Da, Spectrum, United States) and immersed in PBS (pH 5.0 or 7.4). Dtxl release was conducted at 37°C with constant stirring at 100 rpm. At specific time intervals, 1 ml of the sample solution was collected and replaced with equal volume of fresh PBS.

The amounts of Dtxl were analyzed by UPLC-MS/MS (TSQ Quantum Access Max, Thermo Fisher Scientific, United States) with following MS ionization parameters: positive ESI mode; spray voltage, 3500 V; ion source temperature, 300°C; collision energy, 0 eV. The analytes were quantified by using Multiple Reaction Monitoring (MRM) to monitor ion transitions *m*/*z* of 830.2–303.7. Chromatography was performed via Agilent 1100 HPLC with an Ultimate XB-C18 column at the temperature of 40°C and a flow rate of 0.2 ml/min (mobile phase, 0.1% formic acid:methanol = 40:60). The gradient elution was 60% methanol at 0–0.30 min, 60–100% methanol at 0.30–0.50 min, 100% methanol at 0.50–2.00 min, 100–60% methanol at 2.00–2.50 min, and 60% methanol at 2.50–5.00 min.

### Cell Culture

A549, PC3, and DU145 cells were purchased from American Type Culture Collection (ATCC) and cultured by recommended protocols from the manufacturer. Cells were grown in the corresponding medium, supplemented with 10% FBS and 1% penicillin–streptomycin solution, maintained at 37°C and 5% CO_2_.

### Cellular Uptake

A549 cells were seeded in six-well plates (20,000 cells per well) and incubated with 1 ml of complete medium for 24 h. Dil-8P4 NPs at different concentrations were added. At selected time points, cells were washed with cold PBS twice, harvested by trypsinization, centrifuged, and resuspended in 4% formaldehyde, then analyzed by flow cytometer (FACSCalibur, BD, United States).

### Cellular Internalization

A549 cells were seeded in 35-mm dishes (20,000 cells per well) and incubated with 1 ml of complete medium for 24 h. Dil-8P4 NPs were added. At selected time points, cells were washed with cold PBS twice and fixed with 4% formaldehyde at 37°C for 15 min. Subsequently, cells were washed with PBS twice again and stained with LysoTracker green and Hoechst 33342, then observed under an FV3000 confocal laser scanning microscope (CLSM, Olympus, Japan).

### Cytotoxicity

Cytotoxicity was evaluated with AlamarBlue Cell Viability Assay (Thermo Fisher Scientific, United States) against A549, PC3, and DU145 cells. Cells were seeded in 96-well plates (5000 cells per well) and incubated with 0.1 ml of complete medium for 24 h. Cells were incubated with different concentrations of 8P4 NPs, Taxotere, or Dtxl-8P4 NPs. At selected time points, cells were treated according to manufacturer’s protocol using a microplate reader (Synergy4, Bio Tek Instruments, United States).

### Animals

BALB/c mice (male, 4–5 weeks old), nude mice (female, 4 weeks old), and SD rats (male, 200–220 g) were provided by the Laboratory Animal Center of Sun Yat-sen University. This animal study was carried out in accordance with the recommendations of “the guidelines of the Experimental Laboratory Animal Committee of Sun Yat-sen University and the National Institutes of Health’s Guide for the Care and Use of Laboratory Animals.” The animal protocol was approved by the “Experimental Laboratory Animal Committee of Sun Yat-sen University.”

The human pulmonary carcinoma xenograft model was established by subcutaneously injecting A549 cell suspension (2,000,000 cells in medium and Matrigel) into the back region of nude mice. As the volume of xenograft tumor reached ∼100 mm^3^, mice were used for following experiments.

### Pharmacokinetics

SD rats (*n* = 3 per group) were intravenously injected with either (i) PBS, (ii) Taxotere, or (iii) Dtxl-8P4 NPs at a dose of 5 mg Dtxl/kg, respectively. At pre-determined time points, blood was withdrawn from retro-orbital plexus and plasma was collected. Dtxl concentrations were analyzed by UPLC-MS/MS with the same chromatographic condition as described above. Hundred microliters of plasma was mixed with 10 μl of Dtxl, followed by adding 500 μl of methyl tert-butyl ether, vortexed, and centrifuged at 12,000 rpm for 10 min. The supernatant was evaporated and re-constituted with mobile phase. Pharmacokinetic parameters were calculated with Phoenix WinNonlin 6.3 program (Pharsight Corporation, St. Louis, MO, United States).

### Histology Analysis

BALB/c mice (*n* = 5 per group) was intravenously injected with either (i) PBS, (ii) 8P4 NPs, (iii) Taxotere, or (iv) Dtxl-8P4 NPs at a dose of 5 mg Dtxl/kg every 7 days. After 39 days, all the mice were sacrificed and major organs (heart, liver, spleen, lung, kidneys) were excised for hematoxylin and eosin (HE) staining. Slides were observed under a fully automated upright microscope (DM6000 B, Leica, Germany).

### *In Vivo* Antitumor Efficacy

A549 tumor-bearing nude mice (*n* = 5 per group) was intravenously injected with either (i) PBS, (ii) 8P4 NPs, (iii) Taxotere at a maximum tolerated dosage (MTD) of 5 mg Dtxl/kg, (iv) Dtxl-8P4 NPs at a dose of 5 mg Dtxl/kg, or (v) Dtxl-8P4 NPs at a dose of 10 mg Dtxl/kg every 7 days. Body weights and tumor volumes were recorded every 2 days. Tumor volume was calculated as follows:

$$\text{Volume=length}\times {\text{width}}^{2}/2.$$

### HE, IHC, and TUNEL

A549 tumor-bearing nude mice (*n* = 5 per group) was intravenously injected with either (i) PBS, (ii) 8P4 NPs, (iii) Taxotere, or (iv) Dtxl-8P4 NPs at a dose of 5 mg Dtxl/kg every 7 days. After 39 days, tumors were quickly excised for HE and IHC. Slices were incubated with primary antibodies of CD31 and MMP2 (Cell Signaling) and HRP/DAB Detection IHC Kit (Abcam) according to the manufacturers’ instructions.

Terminal deoxynucleotidyl transferase-mediated deoxyuridine triphosphate nick end labeling (TUNEL) was performed according to the instruction of In Situ Cell Death Detection Kit (Roche).

### Statistical Analysis

Results were expressed as mean ± SD and repeated at least three times. Two-tailed Student’s *t*-test was applied to analyze the statistical significance of difference between two groups, one-way analysis of variance (ANOVA) was used for multiple groups. Statistical significance was set at ^∗^*p* < 0.05, ^∗∗^*p* < 0.01, and ^∗∗∗^*p* < 0.001.

## Results

### The Hydrophobic Nature of Phe-PEA Polymers Induced Formation of Dtxl-8P4 NPs with Small Particle Size and High Dtxl Loading

L-Phenylalanine-based poly(ester amide) polymers were prepared by solution polycondensation of monomers I and II at various combinations. By introducing diacid or diol segments with different alkyl chains, the hydrophobic nature of Phe-PEA polymers changed accordingly. As the length of alkyl chain increased, the hydrophobicity as well as loading capacity of polymers enhanced, while the formed NPs tended to possess smaller size, which might be caused by the formation of more dehydrated and compacted cores via hydrophobic force. Due to high drug loading, satisfying entrapment efficiency and reliable stability, Dtxl-8P4 NPs were selected for the following experiments (**Table 3**).

**Table 3:** Dtxl-8P4 NPs at different Dtxl feeding.

Dtxl feeding (%)	Particle size (nm)	Polydispersity index	Zeta potential (mV)	Dtxl loading (%)
10	86.2 ± 0.5	0.125 ± 0.029	-26.39 ± 1.54	8.9 ± 0.9
20	99.7 ± 4.7	0.078 ± 0.011	-28.65 ± 2.98	15.7 ± 1.3
30	172.4 ± 2.0	0.189 ± 0.004	-29.44 ± 2.57	19.6 ± 1.1


**FIGURE 3:**
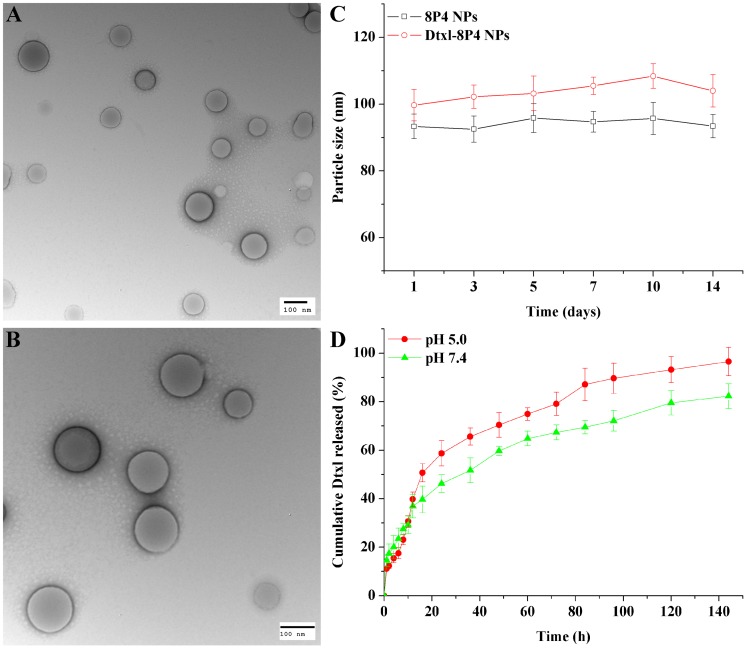
TEM images of **(A)** 8P4 NPs and **(B)** Dtxl-8P4 NPs. **(C)** Particle size of 8P4 NPs and Dtxl-8P4 NPs. **(D)** The cumulative release profiles of Dtxl-8P4 NPs.

Transmission electron microscopy images showed that the loading of a relatively higher amount of Dtxl (∼16 wt%) into 8P4 NPs did not significantly alter the spherical morphology of NPs, but slightly increased their particle size (**Figures 3A–C**).

### Dtxl-8P4 NPs Exhibited Attractive Uptake Kinetics and Strong Cytotoxicity *in Vitro*

*In vitro* release profiles were obtained by representing the percentage of Dtxl released with respect to the amount of drug loaded into NPs. **Figure 3D** demonstrated a sustained-release phase, in which ca. 82.3 and 96.5% of Dtxl were released from Dtxl-8P4 NPs in 144 h at pH 7.4 and 5.0, respectively. This sustained release could mainly result from the erosion and degradation of the components of NPs. Importantly, no burst effect was observed, further confirming that Dtxl incorporated into 8P4 NPs was likely to remain association with NPs and be taken up into cells as the form of particles rather than free drugs.

**FIGURE 4:**
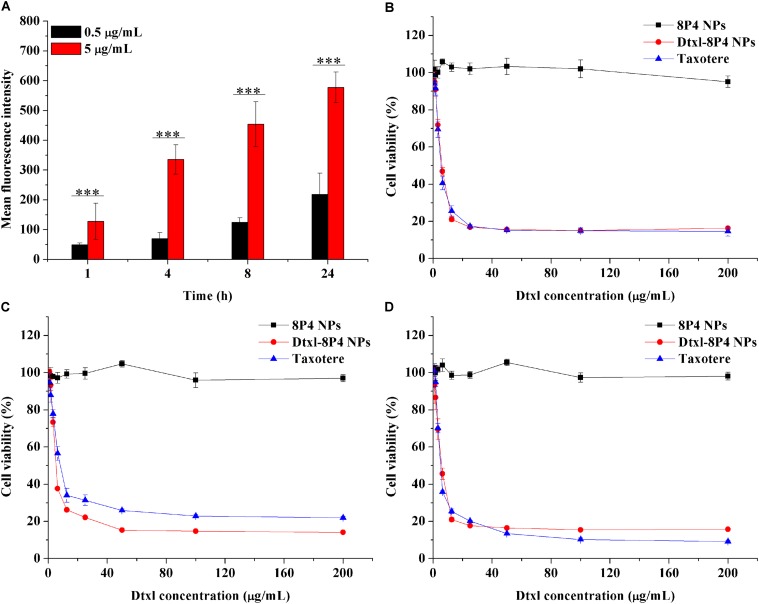
**(A)** Fluorescence intensity of A549 cells incubated with Dil-8P4 NPs. **(B–D)** Cytotoxicity of Dtxl-8P4 NPs and Taxotere against **(B)** A549, **(C)** DU145, and **(D)** PC3 cells after exposure for 48 h.

**FIGURE 5:**
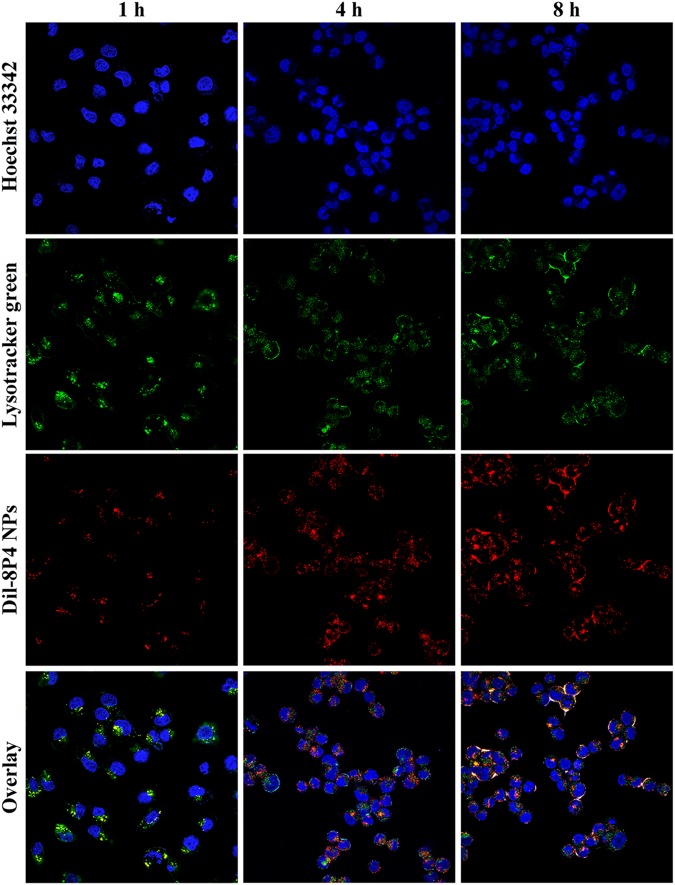
Confocal laser scanning microscope images of A549 cells incubated with 5 mg/ml Dil-8P4 NPs. The nuclei and endosome were stained with Hoechst 33342 (blue) and LysoTracker green (green), respectively (60× objectives).

Cellular internalization of NPs was performed by labeling A549 cells with a specific fluorescent probe, Dil, which was entrapped into 8P4 NPs at a minor amount to minimize the effect on intracellular trafficking. As displayed in **Figure 4A**, prolonging incubation time or increasing incubation concentration of Dil-8P4 NPs resulted in higher internalization, suggesting time- and concentration-dependent manners. After *trans*-membrane transport, Dil-8P4 NPs were found to co-localize with endosomes within 1 h, confirming a relatively fast cellular uptake (**Figure 5**). After that, the red fluorescence was mainly observed in cytoplasm, effectively avoiding the fate of lysosomal degradation. As a next step, *in vitro* cytotoxicity of Dtxl-8P4 NPs was tested against A549, PC3, and DU145 cells by AlamarBlue (**Figures 4B–D**). The viability of cells treated with Dtxl-8P4 NPs did not demonstrate a significant difference compared with Taxotere at low drug levels, but the inhibiting activity of NPs increased at high drug levels. In addition, blank 8P4 NPs without Dtxl had a negligible toxic effect at all test concentrations.

### Dtxl-8P4 NPs Enhanced Therapeutic Effects for A549 Tumors with Less Systemic Toxicity

**FIGURE 6:**
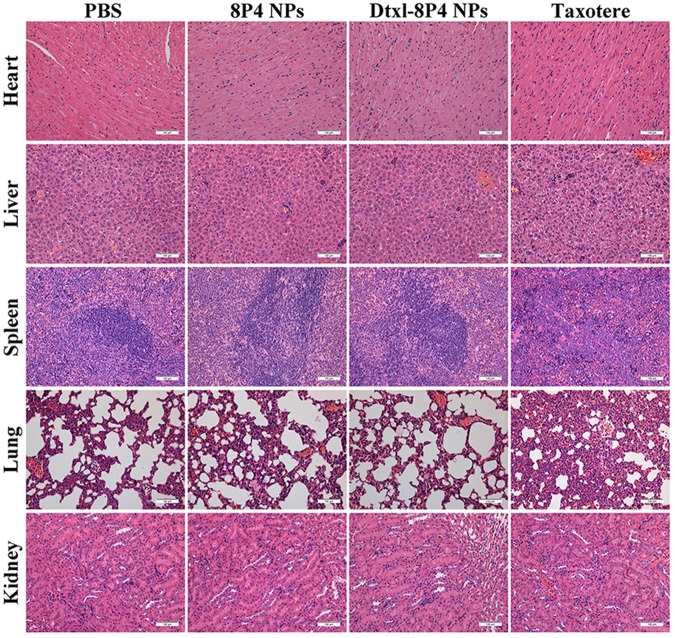
Histopathological analysis of major organs from BALB/c mice (*n* = 5 per group) treated with PBS, 8P4 NPs, Dtxl-8P4 NPs, and Taxotere (20× objectives).

In order to verify whether Dtxl-8P4 NPs impaired major organs, pathological examination was evaluated in healthy BALB/c mice (**Figure 6**). Taxotere caused severe hepatotoxicity with several structural and metabolic changes, i.e., vacuolar degeneration and inflammatory cell infiltration; splenic nodule atrophy; pulmonary hemorrhage; and tubular dilation with flattening of renal epithelium cells. However, no any noticeable histological alternations were captured in Dtxl-8P4 NPs as well as PBS and 8P4 NPs groups, confirming good biocompatibility.

**FIGURE 7:**
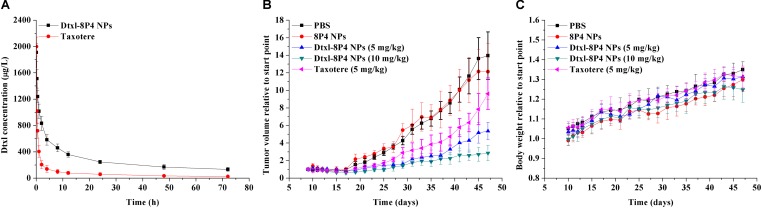
**(A)** Pharmacokinetics after intravenous injection of Dtxl-8P4 NPs and Taxotere at a dose of 5 mg Dtxl/kg (*n* = 3 per group). **(B)** Tumor growth and **(C)** body weight curves of A549 tumor-bearing nude mice (*n* = 5 per group) treated with PBS, 8P4 NPs, Dtxl-8P4 NPs, and Taxotere.

The plasma Dtxl concentrations vs. time profiles were shown in **Figure 7A**. Pharmacokinetics presented the remarkably enhanced retention of Dtxl-8P4 NPs in blood circulation, whereas Taxotere exhibited the rapid elimination from circulation system. Non-compartmental and two-compartmental analysis showed significant changes in pharmacokinetic parameters of Dtxl (**Table 4**). Area under the curve (AUC_0→inf_), area under the first moment curve (AUMC_0→inf_), and mean residence time (MRT_0-inf_) of NPs were 4.7-, 7.6-, and 1.6-fold higher than Taxotere, while clearance (CL) and volume of distribution (*V*_ss_) were reduced by 78.6 and 68.1%, respectively. *t*_1/2_ of distribution and elimination phase was all dramatically extended compared with Taxotere.

**Table 4:** Pharmacokinetic parameters of Dtxl after intravenous injection of Dtxl-8P4 NPs or Taxotere.

Model type	Parameter	Unit	Dtxl-8P4 NPs	Taxotere
Non-compartment model	AUC_0→inf_	h^∗^mg/l	30.069 ± 2.722	6.438 ± 0.549
	AUMC_0→inf_	h^∗^h^∗^mg/l	2034.456 ± 193.682	266.471 ± 0.443
	CL	μl/h/kg	0.166 ± 0.017	0.777 ± 0.039
	*V*_ss_	μl/kg	12.965 ± 2.578	40.705 ± 6.394
	MRT_0→inf_	h	67.660 ± 8.233	41.390 ± 5.081
Two-compartment model	*A*	mg/l	1.287 ± 0.120	2.300 ± 0.287
	α	1/h	1.424 ± 0.331	7.492 ± 2.101
	*B*	mg/l	0.691 ± 0.092	0.800 ± 0.185
	β	1/h	0.039 ± 0.012	0.583 ± 0.186
	*t*_1/2α_	h	0.487 ± 0.113	0.093 ± 0.026
	*t*_1/2β_	h	17.779 ± 5.324	1.190 ± 0.380
	*k*_10_	1/h	0.106 ± 0.023	1.845 ± 0.373
	*k*_12_	1/h	0.834 ± 0.191	3.863 ± 1.214
	*k*_21_	1/h	0.523 ± 0.158	2.366 ± 0.875


To prove the potential of Dtxl-8P4 NPs for tumor growth suppression, a schedule of multiple dosing was applied since day 9 after A549 tumor implantation (**Figures 7B,C**). PBS and 8P4 NPs groups exhibited rapid tumor growth, whereas MTD for weekly dosing of Taxotere significantly delayed tumor growth. In comparison, a better tumor inhibition with sustaining weight gain was observed in mice receiving equal dosing of Dtxl-8P4 NPs. What’s more, the most aggressive treatment with double dosing of chemotherapy suppressed tumor growth for longer but barely induced weight loss, probably due to biocompatible Dtxl-8P4 NPs prevented the random drug release in the body and enhanced therapeutic efficacy of Dtxl.

### Dtxl-8P4 NPs Effectively Suppressed Proliferation, Metastasis, and Apoptosis of Tumors

**FIGURE 8:**
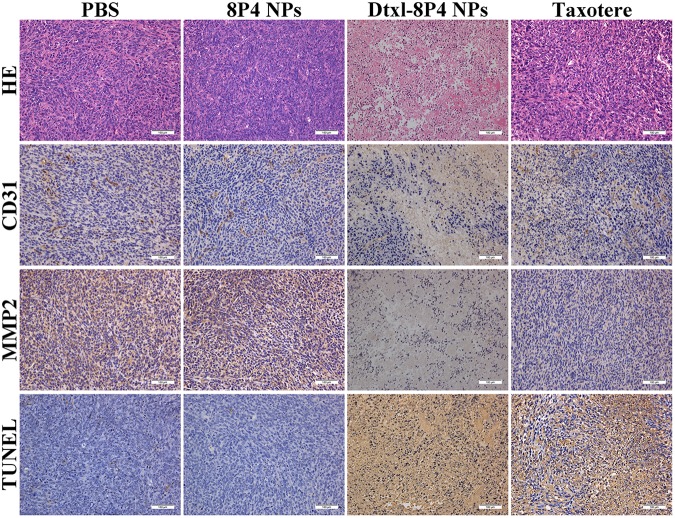
HE staining analysis, CD31 and MMP2 expression and TUNEL examination of tumors from A549 tumor-bearing nude mice (*n* = 5 per group) treated with PBS, 8P4 NPs, Dtxl-8P4 NPs, and Taxotere after a schedule of multiple doses (20× objectives).

The pathology of tumor tissues revealed coincident results (**Figure 8**). For the vigorous growth of tumors in PBS and 8P4 NPs groups, nuclei and cytoplasm presented a blue–pink interlaced image on the whole section. Once tumors underwent apoptosis, nuclei disappeared and cytoplasm became an amorphous mass of necrotic material. The destructed tumor area of Dtxl-8P4 NPs accounted for the highest percentage among all the groups, further revealing the enhanced chemotherapeutic efficiency of NPs. Besides, the histological analysis of proliferation, metastasis, and apoptosis for tumors treated with rounds of chemotherapy was carried out through IHC and TUNEL. Dtxl-8P4 NPs greatly suppressed the expression of CD31 and MMP2, compared with other groups, verifying that tumor proliferation and metastasis were effectively restricted (Giatromanolaki et al., 1997; Bjorklund and Koivunen, 2005; Kim et al., 2009; Jacob and Prekeris, 2015). The administration of PBS or 8P4 NPs caused negligible TUNEL-positive staining, while Dtxl-8P4 NPs resulted in the most remarkable apoptosis of tumors, emphasizing the great efficacy of NPs.

## Discussion

In summary, amino acid-based Phe-PEA polymers were synthesized and formulated with Dtxl to construct Dtxl-loaded Phe-PEA polymer NPs. The hydrophobic nature of polymers contributed to the installation of high hydrophobic payloads. Dtxl-8P4 NPs showed the small particle size ∼100 nm with high loading capacity ∼20 wt%, a low burst effect, and a sustained drug release *in vitro*. Cytotoxicity of Dtxl-8P4 NPs against tumor cells was superior to Taxotere would be attributed to the rapid cellular uptake and effective lysosomal escape. The better antitumor efficacy and less systemic toxicity of Dtxl-8P4 NPs might be attributed to extended blood circulation and high Dtxl loading. Thus, Dtxl-8P4 NPs could be promising as a novel formulation of Dtxl in cancer chemotherapy to fight against NSCLC.

## Author Contributions

XC and LZ conceived and directed the study. YK, ZH, and FX performed syntheses and spectroscopic studies. XC and YK co-wrote the paper. XL and JW oversaw the project and contributed to the execution of the experiments and interpretation of the results. All authors contributed to the characterizations and discussion and reviewed and approved the final paper.

## Conflict of Interest Statement

The authors declare that the research was conducted in the absence of any commercial or financial relationships that could be construed as a potential conflict of interest.

## Supplementary Material

**FIGURE S1 |**^1^H-NMR of Phe-6 (solvent: DMSO-d_6_).
