# Supplementary figures and images for: Significant Suppression of Non-small-cell Lung Cancer by Hydrophobic Poly(ester amide) Nanoparticles with High Docetaxel Loading
Source: Front Pharmacol. 2018 Feb 28;9:118. doi: 10.3389/fphar.2018.00118 (PMC5835838; doi:10.3389/fphar.2018.00118)

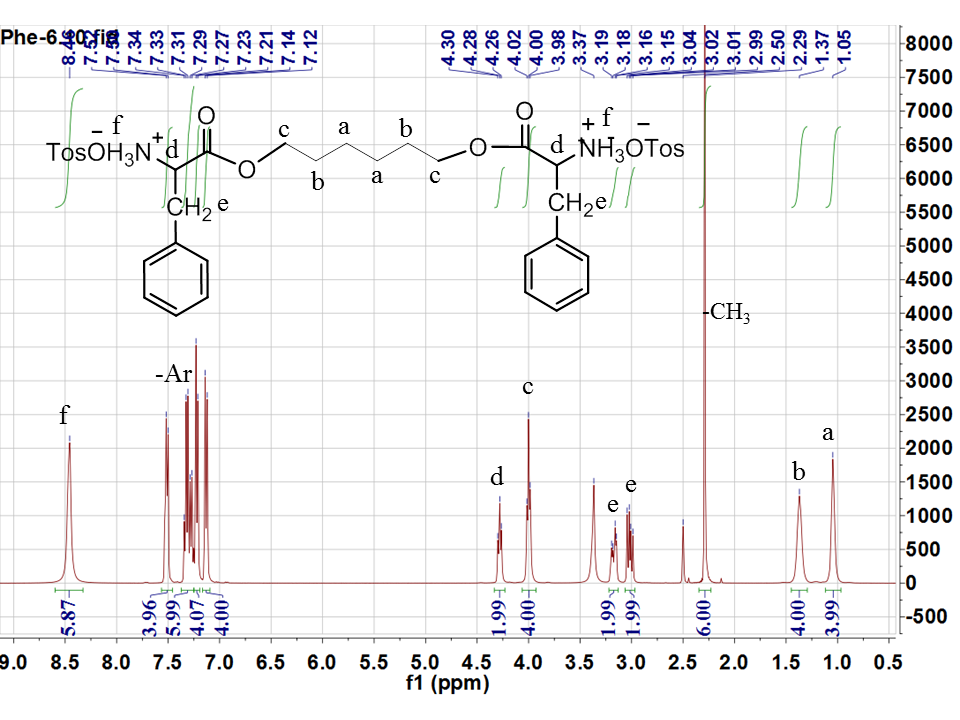

Supplement: Supplementary file 1 [file Image_1.TIF]
